# Magnetic Resonance Imaging of the Neonatal Brain: Modalities, Scoring Systems, and Clinical Applications

**DOI:** 10.3390/bioengineering13080859

**Published:** 2026-07-25

**Authors:** Swathi Ariyapadi, Emily Garavatti, Terrie Inder

**Affiliations:** 1 Division of Newborn Medicine, Rady Children’s Hospital of Orange County, Orange, CA 92868, USA; 2 Center for Perinatal and Infant Research, Rady Children’s Hospital of Orange County, Orange, CA 92868, USA; 3 Department of Pediatrics, University of California, Irvine, CA 92868, USA; 4 Division of Neurology, Rady Children’s Hospital of Orange County, Orange, CA 92868, USA

**Keywords:** magnetic resonance imaging (MRI), neonatal, diffusion-weighted imaging (DWI), diffusion tensor imaging (DTI), volumetric, magnetic resonance spectroscopy (MRS)

## Abstract

Magnetic resonance imaging (MRI) has become the gold standard for evaluating brain injury and development in newborn infants, providing structural, metabolic, and functional information without ionizing radiation. This review comprehensively examines the principal MRI modalities used in current neonatal neuroimaging for a novice/intermediate-level reader—including conventional T1- and T2-weighted imaging, diffusion-weighted imaging (DWI) with apparent diffusion coefficient (ADC) mapping, diffusion tensor imaging (DTI), magnetic resonance spectroscopy (MRS), susceptibility-weighted imaging (SWI), volumetric analysis, arterial spin labeling (ASL), and functional connectivity MRI—with particular attention to their applications in preterm and term populations. Additionally, validated MRI scoring systems for quantifying brain injury severity and predicting neurodevelopmental outcomes are reviewed and summarized. Understanding the technical principles, clinical applications, and limitations of these modalities is essential for optimal interpretation of neonatal brain MRI, and for advancing prognostication and therapeutic decision-making in this vulnerable population.

## 1. Introduction

The neonatal period represents a critical window of brain development characterized by rapid structural and functional maturation. Preterm birth occurs before 37 weeks of gestation and affects approximately 11% of all deliveries worldwide, remaining as the leading cause of neurologic disabilities in children, with nearly 50% of very-low-birth-weight (VLBW) infants suffering some degree of long-term impairment [1]. Term newborn infants may also sustain brain injury from hypoxic–ischemic encephalopathy (HIE), arterial ischemic stroke, infection, or metabolic disorders. Magnetic resonance imaging (MRI) has emerged as the preferred imaging modality for characterizing brain injury and development in both populations, offering the most detailed imaging of the brain while avoiding the radiation risks associated with computed tomography [2,3].

The general theory of MRI is based on the principle that the human body predominantly comprises water, which contains an abundance of hydrogen atoms. When placed within a strong external magnetic field, a small proportion of hydrogen protons align with the field, creating a net magnetization vector. The application of a radiofrequency pulse at the resonant frequency of hydrogen perturbs this alignment, and the subsequent return of protons to their equilibrium state generates detectable signals. The characteristics of this signal recovery, governed by tissue-specific relaxation times, form the basis of image contrast, enabling the differentiation of internal body structures with exquisite soft tissue resolution [3].

Non-invasive neuroimaging methods, such as MRI, are essential to establish links between the brain and behavioral changes in newborns and infants. Specialized methodologies have been developed for data acquisition and processing to address the methodological challenges specific to this population, including sensitivity to motion and the need for age-specific image post-processing tools, as signals and contrasts differ substantially from adult brains and even across varied gestational ages in newborn infants [3,4]. MR sequences must be adapted to the brains of newborns and infants to obtain good, relevant soft-tissue contrast given the small size of cerebral structures and incomplete maturation of tissues [3]. This review examines the principal MRI modalities available for neonatal brain imaging, their applications in preterm and term infants, the validated scoring systems, and the relationship between imaging findings and neurodevelopmental outcomes.

## 2. Conventional MRI: T1-Weighted and T2-Weighted Imaging

### 2.1. Physical Principles

T1-weighted and T2-weighted imaging form the foundation of neonatal brain MRI. These sequences exploit differences in the longitudinal (T1) and transverse (T2) relaxation times of hydrogen protons in different tissues to generate image contrasts [3].

T1 relaxation (spin–lattice relaxation) refers to the signal returned as hydrogen protons realign longitudinally with the main magnetic field following perturbation by a radiofrequency pulse [3]. Tissues with short T1 relaxation times recover rapidly and therefore generate a strong signal, appearing bright or hyperintense on T1-weighted images. In clinical practice, fat, subacute blood products (methemoglobin), and myelinated white matter appear bright on T1-weighted imaging, whereas cerebrospinal fluid (CSF), edema, acute blood and chronic blood products, and unmyelinated white matter appear dark or hypointense [3].

T2 relaxation (spin–spin relaxation) reflects the period during which hydrogen protons remain phase-coherent or synchronized after excitation. Tissues with higher water content maintain phase coherence for longer durations, thereby generating a stronger and more persistent signal that appears bright or hyperintense on T2-weighted images. Conversely, dense tissues with tightly bound protons lose phase coherence rapidly, resulting in signal attenuation and darker areas on imaging. CSF, edema, and unmyelinated white matter appear bright on T2-weighted imaging, while blood products and myelinated white matter appear darker [3].

### 2.2. Unique Characteristics in the Neonatal Brain

The neonatal brain presents unique imaging characteristics due to its high water content and incomplete myelination. For both prematurely born infants and term infants during the first 4 to 6 months of life, unmyelinated white matter demonstrates a lower signal intensity than gray matter on T1-weighted images and a higher signal intensity than gray matter on T2-weighted images—a pattern that is essentially reversed compared with the adult brain [3]. This “reversed” appearance reflects the immature state of myelin development.

**Figure 1:**
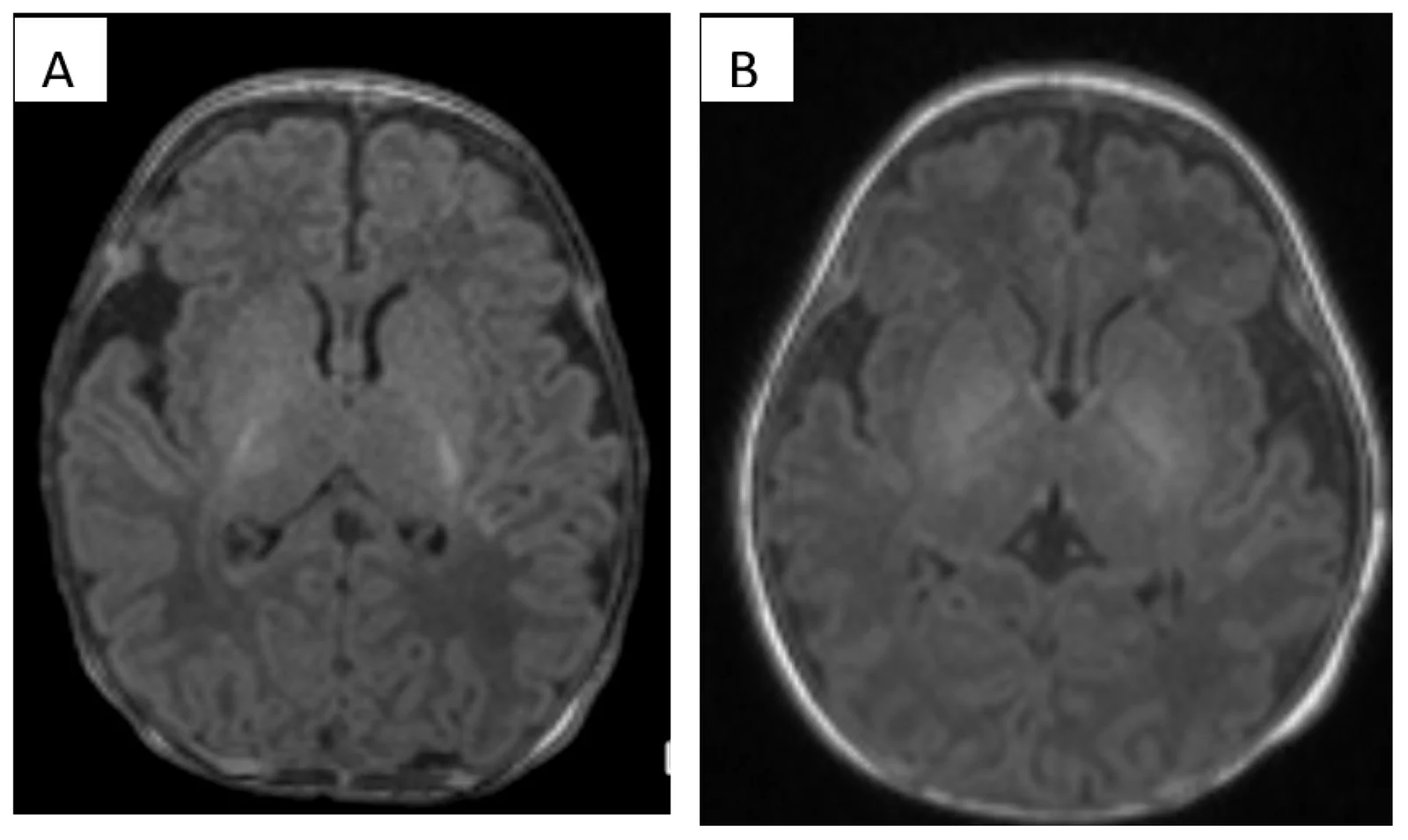
Demonstration of the posterior limb of the internal capsule (PLIC) on T1 imaging (**A**), with brightness indicating the presence of early myelination; conversely, hypointensity (**B**) represents a lack or loss of myelination. (Images are original and obtained from Rady Children’s Hospital’s deidentified patient cases.)

The posterior limb of the internal capsule (PLIC) is among the first structures to myelinate, and it normally demonstrates high T1 signal intensity by term age (Figure 1). Loss of this normal high T1 signal in the PLIC is a reliable and clinically important indicator of severe hypoxic–ischemic injury and correlates strongly with adverse motor outcomes [5,6]. As myelination progresses in a predictable posterior-to-anterior and central-to-peripheral pattern, white matter appears as increased signal intensity on T1-weighted imaging and as decreased signal intensity on T2-weighted imaging [3].

### 2.3. Clinical Applications

T1-weighted imaging is superior for the detection of hyperintensity in the posterolateral putamen and ventrolateral thalamus, the hallmark of the basal ganglia–thalamus injury pattern from profound acute hypoxia, as well as for identifying the loss of normal high-intensity signals in the PLIC [5,7]. The watershed or peripheral pattern of injury from prolonged partial hypoxia involves the cerebral cortex and subcortical white matter, and may be better appreciated using T2-weighted sequences [5].

The comparison of T1 and T2 signal characteristics is also valuable in the identification of hemorrhage at various stages of evolution, the assessment of myelination progression, and the detection of structural malformations [5]. The signal intensity of blood on T1 and T2 sequences evolves predictably as hemoglobin degrades through successive oxidation states, allowing estimation of hemorrhage timing. Subacute hemorrhages characteristically appear as hyperintense in T1-weighted imaging due to the paramagnetic effects of methemoglobin [6,8].

In preterm infants imaged at term-equivalent age (TEA), T1- and T2-weighted imaging can identify white matter injury, germinal matrix hemorrhage, and cerebellar hemorrhage. While there is little consensus regarding which infants qualify for MRI, offering TEA to high-risk infants after discussion with families regarding the limitations and benefits is appropriate and will enhance the recognition and definition of the presence of any brain injury [2]. This may be particularly critical to gain access to limited community resources of therapy services with the documented justification of the presence of brain injury.

### 2.4. Summary

T1- and T2-weighted imaging represent the **clinical standard** for neonatal brain MRI and should be included in all neonatal brain MRI protocols [2,3]. In clinical practice, these sequences are essential for assessing myelination status (including PLIC signal as a prognostic marker), identifying patterns of hypoxic–ischemic injury (basal ganglia–thalamus vs. watershed), staging hemorrhage evolution, and detecting white matter injury and structural malformations at TEA in preterm infants [5,6,8]. T1 and T2 sequences form the backbone of a large majority of validated scoring systems for both term and preterm populations. From a research perspective, ongoing work focuses on optimizing neonatal-specific sequence parameters to improve contrast in the immature brain, developing age-specific signal intensity atlases, and refining the use of T1/T2 signal characteristics as quantitative biomarkers of brain maturity [3,4].

## 3. Diffusion-Weighted Imaging and Apparent Diffusion Coefficient Mapping

### 3.1. Physical Principles

Diffusion-weighted imaging (DWI) measures the random Brownian motion of water molecules in tissue and is uniquely sensitive to the microscopic movement of water, providing information about tissue microstructures and cellular integrity that is not available from conventional sequences [9]. Unlike T1 and T2 imaging, which measure relaxation properties, DWI detects alterations in the diffusional environment of water molecules, enabling early identification of cytotoxic edema and cellular injury [9].

The b-value is a parameter that quantifies the sensitivity of the DWI sequence to water motion; it is proportional to the gradient amplitude, duration, and time interval between gradient pulses and is expressed in units of s/mm^2^. Water molecules with large degrees of motion show signal attenuation at small b-values (50–100 s/mm^2^), while slow-moving water molecules require higher b-values (500–1000 s/mm^2^) for detection. B1000 refers to DWI acquired with a b-value of 1000 s/mm^2^, which is the standard clinical value for brain imaging in the newborn infant [9]. In clinical assessment, tissues with restricted water diffusion (due to cellular swelling or ischemia) will appear hyperintense or bright, while other areas of normal or increased diffusion may appear dark.

The apparent diffusion coefficient (ADC) is a quantitative measure calculated from DWI data that represents the slope of signal decay across different b-values, with the critical benefit of eliminating T2 shine-through that can otherwise confound DWI interpretation [9]. Following brain injury resulting in cellular necrosis, ADC is initially reduced when cytotoxic edema leads to diffusion restriction in injured regions. True restricted diffusion appears as hyperintensity on DWI, with corresponding hypointensity (low ADC values) on ADC mapping. Low ADC values have been associated with increased death or neurodevelopmental impairment (NDI) in neonates affected by ischemic encephalopathy [5].

### 3.2. Clinical Applications and Timing

**Figure 2:**
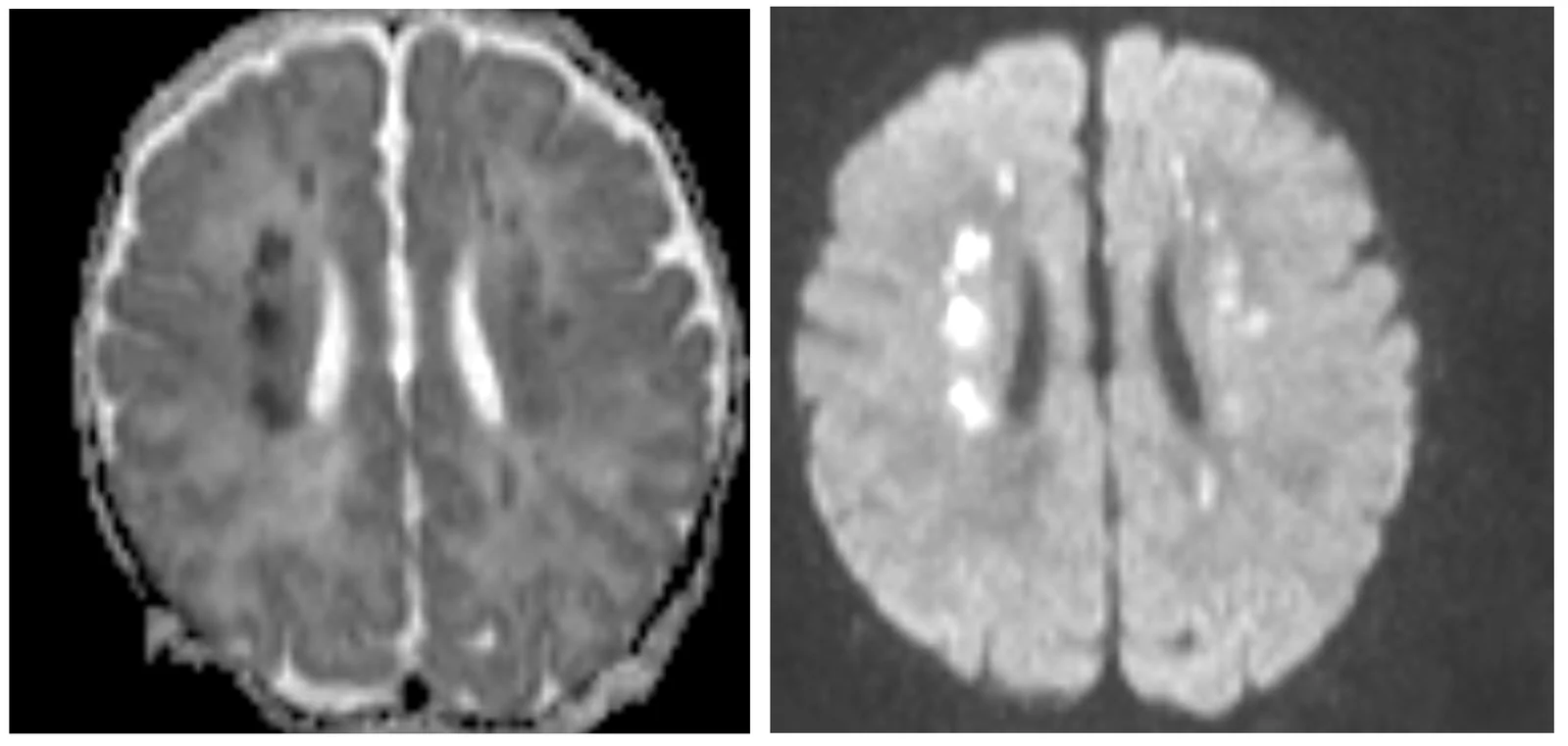
Images demonstrating restricted diffusion in the distribution of the watershed zone as a result of ischemic injury, with hyperintensity in the diffusion-weighted image (DWI, (**left**)) and corresponding hypointensity in the apparent diffusion coefficient (ADC, (**right**)). Images are original and obtained from Rady Children’s Hospital’s deidentified patient cases.

**Figure 3:**
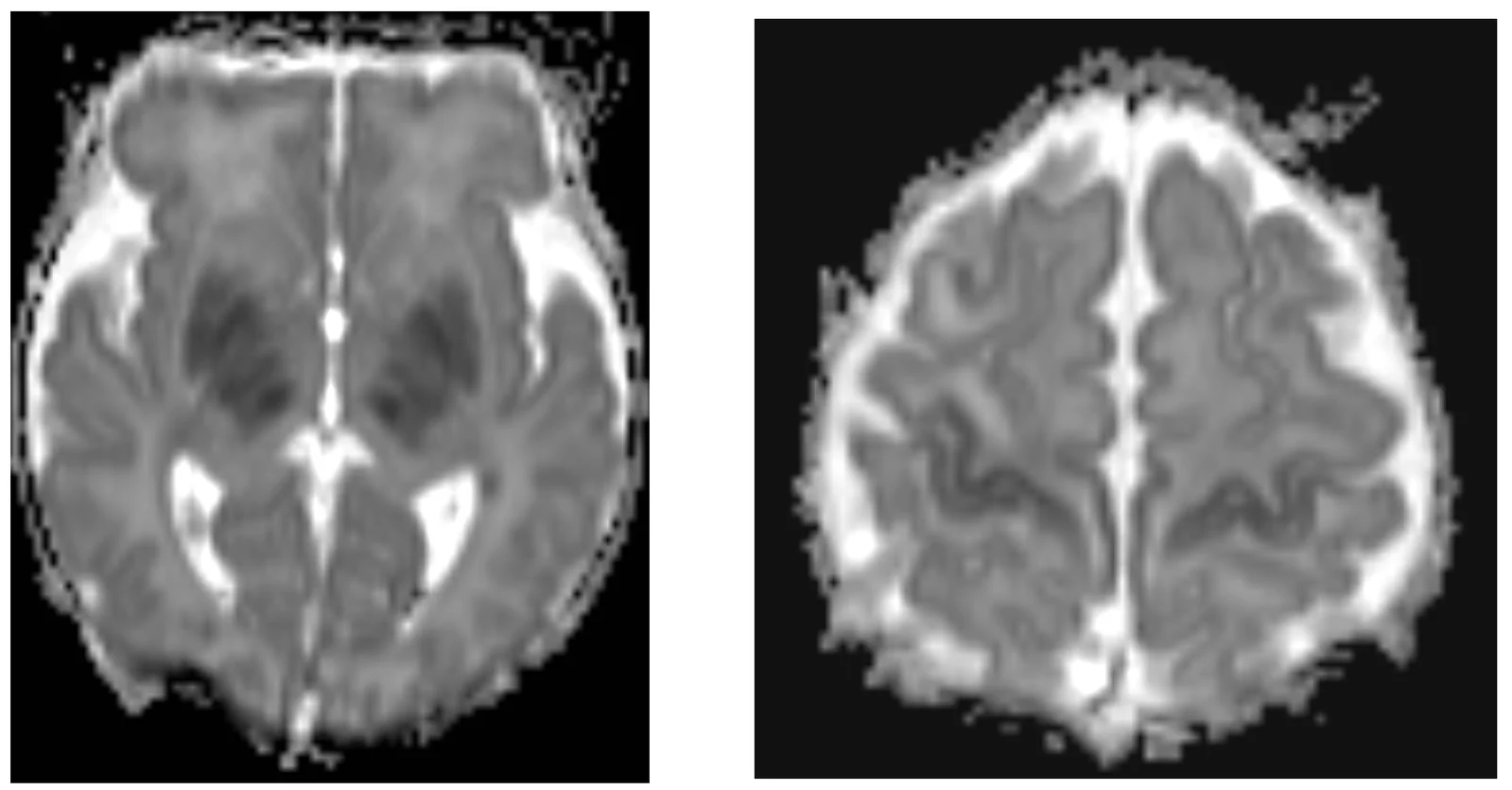
Restriction in the apparent diffusion coefficient (ADC) mapped on diffusion-weighted imaging shows as dark regions, representing abnormality in the basal ganglia (**left**) and perirolandic region (**right**) related to ischemic injury. Images are original and obtained from Rady Children’s Hospital’s deidentified patient cases.

DWI enables early detection of injury, often within 12 h of the insult, before T1 and T2 changes become apparent (Figure 2 and Figure 3) [5,9]. Peak DWI abnormalities, particularly those related to hypoxic–ischemic changes, are most often observed on days 2 to 5 from injury, representing the optimal window for injury detection [6]. Days 5 to 7 often mark a period of pseudonormalization in infants who have not undergone therapeutic hypothermia, while pseudonormalization is delayed to days 8 to 12 in infants who have been cooled [6,10]. This pseudonormalization phenomenon, in which ADC values return to normal despite permanent tissue damage, represents a critical diagnostic pitfall that limits the utility of DWI to the first week after injury. Early MRI to assess DWI changes is appropriate in cases that need early prognostication. It is important for the clinician to be aware that the optimal use of DWI should be immediately after rewarming to allow maximal sensitivity to restricted diffusion. If the diffusion acquisition is delayed after day 5, or if the injury is suspected to be remote or earlier than birth, then the DWI may be falsely negative.

The optimal timing of DWI in preterm infants is less well-known, with many protocols focusing on TEA imaging to assess white matter or cystic lesions and other signs of remote injury. There is discussion regarding the future possible utility of earlier MRI in preterm infants, with the possibility of an added benefit that allows the identification of infants with injury who may benefit from immediate neuroprotective strategies. In these cases, DWI, along with other MRI modalities, is useful in comprehensive evaluation of brain injury, healing, and prognosis.

### 3.3. Prognostic Value

In term infants, lower ADC values in the basal ganglia during the first 7 days of life predict adverse neurologic outcomes [5]. Injuries to the PLIC and basal ganglia are associated with motor deficits, while combined injury to the PLIC, diffuse basal ganglia, and peripheral hemispheric gray and white matter is associated with death, hearing and visual impairments, and severe cerebral palsy [5].

In preterm infants, DWI can detect punctate white matter lesions and periventricular leukomalacia, both of which carry significant prognostic implications [11]. ADC values, especially in the frontal white matter, can effectively evaluate the potential for neurodevelopmental disorders in preterm infants, which can in turn facilitate early therapeutic intervention [3]. Notably, moderate–severe and anterior white matter injury from early-life MRI has been associated with cognitive delays (OR 3.28) and motor delays (OR 3.02) at 36 months, suggesting that early-life MRI may represent a more optimal timepoint for assessing white matter injury in very preterm infants [12].

### 3.4. Summary

DWI with ADC mapping is a clinical standard that should be included in all neonatal brain MRI protocols, particularly for the evaluation of neonatal encephalopathy [6,9]. Its primary clinical role is the early detection of acute ischemic injury, often before conventional sequences become abnormal and after the first 12 h of life. Quantitative ADC values in the basal ganglia provide robust prognostic information for death and neurodevelopmental impairment [5,9]. Clinicians must be aware of the pseudonormalization window (days 5–7 without cooling; days 8–12 with cooling), which represents a critical diagnostic pitfall [6,10]. In preterm infants, DWI detects punctate white matter lesions and periventricular leukomalacia with prognostic significance if undertaken in the acute period [11,12]. From a research perspective, ADC thresholds for predicting specific outcomes are continuing to be refined, the optimal timing of DWI in preterm populations is under investigation, and the potential role of early DWI (prior to TEA) for identifying preterm infants who may benefit from neuroprotective strategies is an active area of study [9,12].

## 4. Diffusion Tensor Imaging

### 4.1. Physical Principles

Diffusion tensor imaging (DTI) extends DWI by measuring water diffusion in multiple directions to characterize the three-dimensional diffusion properties of tissue. The diffusion tensor is a mathematical representation of diffusion magnitude and directionality, allowing detailed assessment of white matter microstructure and connectivity [9].

Key DTI metrics include:

- Fractional anisotropy (FA): Measurement of the degree of directional preference in diffusion (range 0–1), with higher FA indicating more organized, myelinated white matter.
- Mean diffusivity (MD): Average diffusion across all directions, reflecting overall tissue water content and cellularity.
- Axial diffusivity (AD): Diffusion along the principal axis, parallel to axonal fibers.
- Radial diffusivity (RD): Diffusion perpendicular to the principal axis, which is particularly sensitive to myelination status.

### 4.2. Applications in Brain Development

DTI is of particular use in understanding brain development in preterm infants. Maturational changes in diffusivity measures between 30 and 40 weeks postmenstrual age generally show a central-to-peripheral and posterior-to-anterior gradient of change, consistent with known patterns of brain maturation [3]. The largest increases in FA occur in central brain regions, reflecting progressive myelination, while cortical brain regions show a decrease in FA, reflecting dendritic arborization and increasing complexity [3].

### 4.3. Preterm vs. Term Differences

Systematic reviews have demonstrated widespread differences in diffusion measures in preterm infants at TEA compared with full-term newborn infants, with more marked differences in very preterm infants [3]. These abnormalities suggest changes in white matter microstructure that may underpin the increased risk for neurodevelopmental disability seen in preterm infants in later life. The corpus callosum is a region of particular interest, showing diffusion abnormalities in both early and moderate–late preterm groups across multiple studies; DTI may therefore serve as a useful prognostic tool for neurodisability in preterm neonates [13].

In neonatal encephalopathy, DTI provides various scalar measures that represent tissue properties affected by the microscopic cellular and extracellular environment. Previous studies have demonstrated widespread alteration in DTI measurements in severe cases of HIE and more localized changes in neonates with mild-to-moderate HIE [14]. Measurements in the corpus callosum, thalamus, basal ganglia, corticospinal tract, and frontal white matter have demonstrated excellent ability to predict severe neurological outcomes [14].

### 4.4. Diffusivity and Tensor Maps

DTI data can be visualized through diffusivity maps and tensor maps that provide complementary information about white matter architecture. Diffusivity maps display scalar values (FA, MD, AD, RD) at different points in the brain, enabling quantitative comparison across brain regions and between subjects. These maps reveal the spatial distribution of microstructural properties, and can be used to track maturational changes over time or to identify regions of injury [14].

Tensor maps show the shape and direction of water movement at each point, displaying these as three-D ellipsoids. In highly organized white matter tracts, tensors appear elongated along the fiber direction (high anisotropy), whereas in gray matter or regions of crossing fibers, tensors appear more spherical (low anisotropy). Color-coded FA maps use a directional color convention (red for left–right, green for anterior–posterior, blue for superior–inferior) to visualize the principal diffusion direction at different foci, providing an intuitive representation of white matter tract orientation [14].

Advanced diffusion kurtosis imaging (DKI) is a more advanced technique that goes beyond standard DTI by capturing more complex patterns of water movement. It provides additional measures, such as axonal water fraction, fiber dispersion, and extra-axonal diffusivity, which can detect aspects of brain maturation not visible by DTI alone, particularly with changes within axons and in fiber organization during the neonatal period [3].

### 4.5. Advanced Diffusion Techniques

Neurite Orientation Dispersion and Density Imaging (NODDI) provides more specific microstructural information than DTI by modeling intracellular, extracellular, and CSF compartments separately. NODDI enables assessment of neurite density and orientation, offering insights into axonal and dendritic development that are not available from conventional DTI metrics [3].

Constrained Spherical Deconvolution (CSD) enables multiple fiber populations within an imaging region to be resolved, allowing delineation of areas of with fiber-crossing, such as the arcuate fasciculus and cerebellar-cortical pathways. Fixel-based analysis (FBA) represents another recent advance that enables white matter microstructure to be assessed in detail at the level of individual fiber populations within a voxel [3].

### 4.6. Summary

DTI is currently used primarily as a research tool in neonatal neuroimaging, with growing potential for clinical translation [3,13,14]. Its principal research applications include characterizing white matter microstructural development across gestational ages; identifying diffusion abnormalities that distinguish preterm from term-born infants (particularly in the corpus callosum); and predicting neurodevelopmental outcomes in both HIE and preterm populations through quantitative metrics, such as FA and MD, in the basal ganglia, thalamus, and corticospinal tracts [13,14]. DTI is not a part of routine clinical MRI protocols due to longer acquisition times, susceptibility to motion artifacts, and the need for specialized post-processing. Advanced extensions, including DKI, NODDI, CSD, and FBA, remain in the research domain, offering increasingly specific microstructural information. All of these applications require further validation before clinical adoption [3].

## 5. Magnetic Resonance Spectroscopy

### 5.1. Physical Principles

Proton magnetic resonance spectroscopy (MRS) allows for in vivo quantitative analysis of brain metabolites, providing biochemical information that complements structural imaging [5,9]. MRS detects signals from hydrogen atoms in specific metabolites based on their unique chemical shift frequencies, generating a spectrum of peaks that correspond to different metabolic compounds.

Key metabolites assessed in neonatal MRS include [9]:

- N-acetylaspartate (NAA): A marker of neuronal integrity and viability; reduced NAA reflects neuronal loss or dysfunction.
- Lactate (Lac): A marker of anaerobic metabolism, which becomes elevated after hypoxic–ischemic injury, though this is often transient.
- Choline (Cho): A marker of membrane turnover and myelination.
- Creatine (Cr): A measure of energy metabolism, often used as an internal reference for metabolite ratios.
- Glutamate and glutamine (Glx): Markers of excitotoxicity, becoming elevated in the acute phase of HIE.
- Myo-inositol (mI): A glial marker; the mI/NAA ratio has shown predictive value for outcomes.

### 5.2. Clinical Applications and Prognostic Value

MRS is particularly valuable in neonatal encephalopathy because it can detect injury earlier than conventional MRI. Findings from MRI without spectroscopy could be normal for as long as 24 h after an acute hypoxic–ischemic event, but MRS or DWI detects early acute events [5]. When clinicians add MRS to standard MRI, the scanning time increases by only 6 to 7 min and may improve the predictive value of the scan [5].

The lactate/NAA ratio from the basal ganglia and thalamus provides robust prognostic information regarding 2-year neurodevelopmental outcomes [9]. The landmark MARBLE study demonstrated that thalamic NAA concentration, measured within 14 days after birth, accurately predicted adverse neurodevelopmental outcomes 2 years later [9]. The lactate/NAA peak-area ratio displayed a sensitivity of 88% and specificity of 90% for predicting adverse outcomes; however, the prognostic accuracy of this ratio appears to be primarily driven by NAA concentration, reflecting neuronal loss rather than ongoing anaerobic metabolism [9].

The combination of MRI scoring with ADC values or lactate/NAA ratios in the basal ganglia has a better association with outcome of term newborn infants with HIE than MRI alone [9]. Proton MRS should be a routine component of clinical MR protocols for infants with neonatal encephalopathy [8,9].

### 5.3. Clinical Applications and Prognostic Value

MRS is recommended as a routine clinical component of MRI protocols for infants with neonatal encephalopathy, adding only 6–7 min to scan times while substantially improving prognostic accuracy [5,8,9]. The lactate/NAA ratio from the basal ganglia and thalamus provides the most robust prognostic biomarker, with 88% sensitivity and 90% specificity for predicting adverse 2-year neurodevelopmental outcomes, and the combination of MRI scoring with MRS metrics outperforms MRI scoring alone [9]. Clinically, MRS is particularly valuable for detecting injury alongside diffusion imaging earlier than conventional sequences to assist in early goals-of-care discussions. It can also provide additional prognostic information at later imaging timepoints. From a research perspective, MRS is being investigated as a surrogate endpoint in neuroprotection trials, and additional metabolites (glutamate/glutamine, myo-inositol) are under study for their prognostic value in both term and preterm populations [9].

## 6. Susceptibility-Weighted Imaging

### 6.1. Physical Principles and Clinical Applications

Susceptibility-weighted imaging (SWI) enhances image contrast by exploiting susceptibility differences between tissues. SWI is especially sensitive to deoxygenated blood, iron deposition, and calcium, making it particularly useful for detecting hemorrhage, with significantly higher detection rates than T1-weighted imaging, and it can also provide high-resolution delineation of cerebral venous architecture [8].

In newborn infants, SWI provides valuable additional diagnostic and prognostic information for a wide spectrum of neurological disorders. The positive detection rate of intracranial extracerebral hemorrhage by SWI is higher than that of T1-weighted imaging, and asymptomatic neonatal intracranial hemorrhage may be a common complication of the birth process that SWI can identify [8]. SWI may also be more sensitive than T1-weighted imaging for detecting damage in the globus pallidus in kernicterus, as it lacks the disadvantage of the T1 sequence where early myelin confers a high signal that can mask injury [8]. Additionally, SWI provides superior visualization of the cerebral venous system, useful for detecting venous thrombosis and developmental venous anomalies. A European multicenter collaboration proposed a standardized MRI protocol for CHD neonates that includes T1, T2, DWI, SWI, and MR venography to capture the full spectrum of ischemic, hemorrhagic, and thrombotic lesions [15].

### 6.2. Summary

SWI is recommended as a standard acquisition in clinical neonatal brain MRI protocols, particularly when hemorrhage detection is a priority [8,15]. Its clinical utility lies in its superior sensitivity for detecting intracranial hemorrhage compared to T1-weighted imaging, as well as its identification of globus pallidus injury in kernicterus and high-resolution visualization of cerebral venous architecture for detecting venous thrombosis [8]. SWI has been incorporated into standardized multicenter MRI protocols for CHD neonates alongside T1, T2, DWI, and MR venography [15]. From a research perspective, the full prognostic significance of SWI-detected microhemorrhages in neonates, including asymptomatic birth-related hemorrhage, remains under investigation, and normative SWI reference data for the neonatal population are still being established [8].

## 7. Volumetric MRI Analysis

### 7.1. Principles and Methods

Volumetric MRI analysis uses three-dimensional imaging and computational algorithms to quantify total brain volume and regional volumes of specific structures. The neonatal brain can be divided into multiple tissue classes, including cortical gray matter, unmyelinated white matter, myelinated white matter, CSF, basal ganglia, thalamus, brainstem, and cerebellum [16,17]. Neonatal MR-based brain volumes can serve as objective, quantitative, and reproducible surrogate parameters of early brain development [16,18].

### 7.2. Developmental Trajectories

Quantitative MRI studies have demonstrated that total brain tissue volume increases linearly at a rate of approximately 22 mL/week between 28 and 40 weeks postconceptional age or TEA [16,19]. The total gray matter shows a linear increase of approximately 1.4% or 15 mL in absolute volume per week, reflecting primarily a fourfold increase in cortical gray matter from 28 weeks to TEA. Unmyelinated white matter is the most prominent brain tissue class in preterm infants younger than 36 weeks. Between 35 and 41 weeks, there is an abrupt fivefold increase in absolute volume of myelinated white matter, reflecting the rapid myelination during this critical developmental period [18,20].

### 7.3. Preterm Brain Dysmaturation

**Figure 4:**
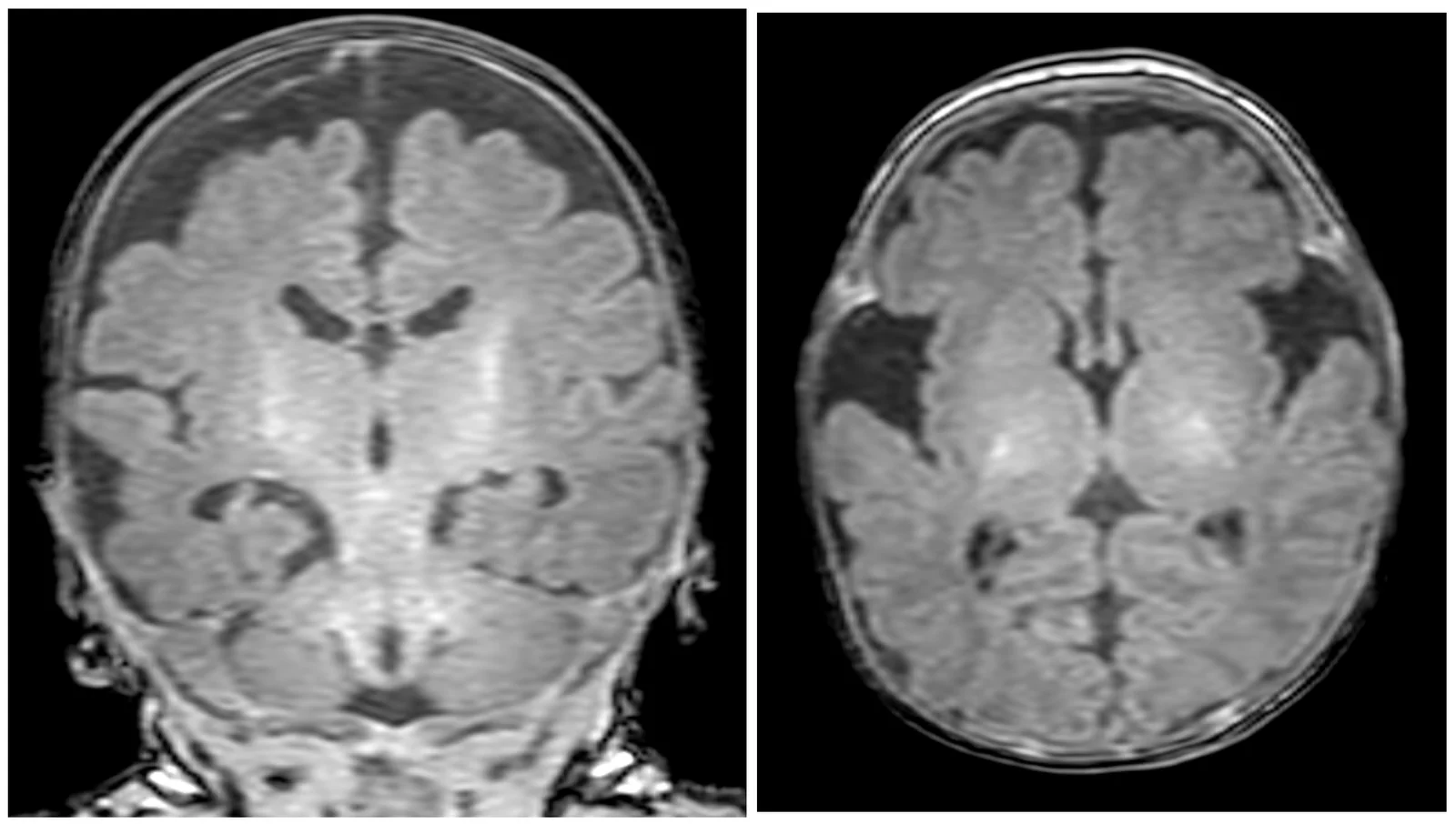
Preterm birth has been shown to be associated with prominent extra-axial spaces, thinning of the corpus callosum, and under-opercularization of the sylvian fissures, as demonstarted in these coronal T1-weighted (**left**) and axial T1-weighted images (**right**). Images are original and obtained from Rady Children’s Hospital’s deidentified patient cases.

Preterm infants at term have been demonstrated to have smaller total cerebral volumes, with reductions in both white matter and gray (cortical and subcortical) matter volumes, reduced cortical surface areas, and larger ventricular volumes compared to term-born controls (Figure 4) [15].

Surface-based MRI measures show decreased cerebral cortical surface area and cortical gyrification in preterm infants at TEA, with reduced sulcal depth particularly in the temporal and frontal regions. These structural alterations persist into childhood and correlate with the cognitive, language, and attention deficits commonly observed in children born preterm, supporting the concept of brain dysmaturation as distinct from but potentially overlapping with brain injury [20].

Early brain morphometrics from the neonatal MRI (acquired at 29–35 weeks postmenstrual age) can predict motor and cognitive outcomes at 2 years corrected age in very preterm infants, with associations identified for cortical gray matter volumes, cortical thickness, and sulcal depth (AUC 0.86 for both motor and cognitive adverse outcomes) [16].

### 7.4. Summary

Volumetric MRI analysis is currently used **primarily as a research tool**, though it has significant potential for clinical translation as a quantitative biomarker of brain development and dysmaturation [16,17,18]. Research applications include quantifying the trajectory of brain growth (approximately 22 mL/week between 28 and 40 weeks), characterizing the fourfold increase in cortical gray matter and the fivefold increase in myelinated white matter during the preterm-to-term transition, and identifying volumetric reductions in preterm infants at TEA compared with term controls [16,19,20]. Early brain morphometrics have demonstrated a strong predictive value for motor and cognitive outcomes at 2 years (AUC 0.86) [16]. Clinical translation is limited by the need for specialized computational algorithms, labor-intensive segmentation, and the absence of widely accepted normative reference ranges—although automated deep-learning-based segmentation pipelines are beginning to address these barriers [21].

## 8. Arterial Spin Labeling Perfusion MRI

### 8.1. Physical Principles

Arterial spin labeling (ASL) is a non-invasive MRI technique for assessing cerebral perfusion that uses magnetically labeled water protons from arterial blood as an endogenous diffusible tracer, eliminating the need for exogenous contrast agents [22,23]. This characteristic makes ASL particularly attractive for neonatal imaging, where gadolinium administration is generally avoided. ASL provides quantitative cerebral blood flow (CBF) measurements, enabling comparative analysis across longitudinal studies [22,23]. 

### 8.2. Clinical Applications

Abnormal brain perfusion is a key mechanism underlying neonatal brain injury, and understanding perfusion changes in high-risk newborn infants is essential for improving therapeutic strategies [24]. In term neonates with HIE, ASL can demonstrate hyperperfusion in the basal ganglia and thalami during the reperfusion phase, which correlates with injury severity [24,25]. In preterm infants, CBF positively correlates with postmenstrual and postnatal age, and sex-related hemodynamic variations have been observed [26]. The highest CBF in healthy infants has been observed in the basal ganglia and thalamus, consistent with the high metabolic demand of these structures [27].

### 8.3. Summary

ASL is an emerging modality that is **transitioning from research toward clinical application** in neonatal neuroimaging [22,23,24]. Its primary advantage is the ability to quantify cerebral blood flow without exogenous contrast agents, making it particularly suitable for neonates in whom gadolinium is generally avoided [22,23]. Clinically, ASL can identify hyperperfusion in the basal ganglia and thalami during the reperfusion phase of HIE, which correlates with injury severity, and it can characterize normal perfusion patterns across gestational ages [24,25,27]. From a research perspective, the optimization of ASL parameters for neonatal brains (including multi-delay techniques), establishment of age-specific normative CBF values, and investigation of perfusion changes as early biomarkers of injury and predictors of outcome are active areas of investigation [23,26,27]. ASL is not yet a standard component of routine clinical neonatal MRI protocols at most centers.

## 9. Functional Connectivity MRI

### 9.1. Physical Principles

Functional connectivity MRI (fcMRI) utilizes spontaneous, low-frequency (0.1 Hz), coherent fluctuations in blood oxygen level-dependent (BOLD) signals to identify networks of functional cerebral connections. This technique can be performed during natural sleep making it well-suited for neonatal populations. The resting-state networks (RSNs) identified through fcMRI reflect the evolving cerebral structural architecture, presumably driven by varied genetic and environmental influences [3].

### 9.2. Summary

Functional connectivity MRI remains a research tool in neonatal neuroimaging [3]. Its ability to identify resting-state networks during natural sleep makes it well-suited for neonatal populations, and it has provided important insights into the development of functional brain architecture across gestational ages. However, fcMRI is not currently used in clinical practice for neonatal populations due to the complexity of data acquisition and analysis, the lack of validated clinical applications, and the absence of normative reference data. Research efforts are focused on characterizing the emergence of resting-state networks in preterm and term infants, understanding how early brain injury disrupts functional connectivity, and determining whether fcMRI metrics can serve as prognostic biomarkers for neurodevelopmental outcomes [3].

## 10. MRI Scoring Systems

### 10.1. Rationale

Imaging assessment and interpretation has historically been limited by the subjectivity of an individual analyzer or radiologist, which has led to the creation of more objective scoring systems of injury. These systems aim to better standardize data interpretation and facilitate understanding of short- and long-term outcomes in infants with different degrees of injury [9].

### 10.2. Scoring Systems for Term Infants with Neonatal Encephalopathy

In term infants being evaluated for hypoxic–ischemic encephalopathy, several scoring systems exist, including the Barkovich, Rutherford, NICHD NRN, Weeke, and Trivedi systems (Table 1). A systematic review of 16 studies encompassing 1925 participants found that all MRI scoring systems have similar predictive accuracies, and simpler systems, such as the NICHD, performed on par with their more complex counterparts [28]. Most scoring systems in the term population include some variation in severity scoring of injury to the deep gray matter, white matter, cerebellum, cortex, and important myelination pathways through assessment of T1, T2, and DWI, and many have long-term neurodevelopmental correlates [7,9,29].

While severe injury is best assessed by such systems, there remains a significant challenge in evaluating and understanding mild and moderate injury. Infants with mild or moderate MRI brain injury, when using two of the scoring systems, were shown to have similar Bayley-III cognitive, language, and motor scores as infants with no injury, underscoring the need for cautious counseling regarding a range of possible outcomes [7,30]. 

However, it is important to recognize that several of the more recent scoring systems, based on the Weeke scoring system, have greater comprehensive definitions of the nature of injury in the term newborn, with higher predictive values than the scoring systems described above. It is also critical to recognize that outcomes beyond cerebral palsy or significant neurodevelopmental disability are important, particularly impaired cognition and behavioral outcomes. Most recently, abnormalities in the mamillary bodies have been shown to indicate specific ischemic injury in term newborn, and have shown predictive power for processing, memory, and overall cognition at and past 7 years of age [31,32].

### 10.3. Scoring Systems for Preterm Infants

Evaluation of preterm brain injury is fundamentally different from term injury due to the distinct nature and etiology of injury in this population. Germinal matrix hemorrhage, cerebellar hemorrhage, and white matter injury represent the principal forms of brain injury in preterm infants, related to immaturity and differences in vascular regulation to high-risk brain structures [2,33]. Depending on the type of expected injury, various scoring systems exist to best evaluate the trajectory of injury in the neonatal brain (Table 2).

### 10.4. The Woodward Scoring System

The Woodward scoring system was the first system to be developed to formally assess white matter injury (WMI) and gray matter injury (GMI) separately. The WMI score (range 5–15) sums subscores for white matter signal abnormality, periventricular white matter volume loss, cystic abnormalities, ventricular dilatation, and thinning of the corpus callosum. The GMI score (range 3–9) sums subscores for cortical abnormalities, gyral maturation quality, and subarachnoid space size [34]. In this study, moderate-to-severe white matter abnormalities on TEA MRI were associated with cognitive delays, psychomotor delays, cerebral palsy, and neurosensory impairment at 2 years corrected age [34].

### 10.5. The Kidokoro System

This first scoring system was then expanded to create the most widely used and comprehensive scoring system for preterm brains at TEA, known as the Kidokoro system, which involves an organized assessment of white matter, cortical gray matter, deep gray matter, and the cerebellum, generating a global brain abnormality score (GBAS) [33,35]. This system included systematic measurements of brain regions to define loss of brain volume in addition to the presence of injury.

This scoring system is also validated for use in early MRI (32 weeks gestational age), which poses a unique additional benefit, though early scores are consistently higher than TEA scores primarily due to the myelination delay item [36]. When this item is excluded, the discrepancy between early and TEA scores is substantially reduced [36]. The GBAS was associated with cognition, motor skills, and behavior at 2 years of age, but this diminished by age 10 years where the birth weight and level of maternal education were predictive of cognitive outcomes, suggesting that environmental factors become increasingly important as children grow older [33]. Higher global brain, cerebral white matter, and deep gray matter abnormality scores were related to poorer IQ, spelling, math computation, and motor function at 7 years of age, with moderate–severe global abnormality associated with a reduction in IQ of 6.9 points independent of clinical and social confounders [37]. Prolonged mechanical ventilation (>7 days) and parenteral nutrition (>21 days) have been identified as independent perinatal risk factors for higher GBAS [35]. Importantly, a reduction in GBAS from early to TEA MRI correlated with higher motor scores at 24 months, suggesting that longitudinal scoring may capture recovery trajectories [36].

### 10.6. Steven Miller White Matter Injury Scoring

The Miller classification system is a semi-quantitative grading scale applied to T1-weighted MRI sequences on which punctate WMI appears as foci with high signal intensity. The Miller classification stratifies injury into three grades based on lesion number, size, and extent of hemispheric involvement [38,39]. The system is optimally applied on early-life MRI obtained between 30 and 34 weeks postmenstrual age, when punctate lesions are most conspicuous on T1-weighted imaging [12].

Moderate-to-severe WMI (grades 2–3) is associated with an elevated risk of motor, cognitive, and language impairment at 18 months and 4.5 years corrected age [38]. Even minimal WMI (grade 1) is not entirely benign, as small numbers of punctate lesions have been associated with altered brain development and adverse motor outcomes [40]. Quantitative analyses have confirmed a continuous, dose-dependent relationship between lesion volume and adverse white matter microstructural development [39]. 

Emerging evidence indicates that lesion location provides prognostic information beyond severity grading alone; anterior WMI is more strongly associated with motor and cognitive impairment than posterior WMI [12,41]. Additionally, while early-life MRI best captures acute lesion burden, MRI at TEA is better suited for assessing secondary sequelae such as white matter volume loss and delayed myelination [12].

The categorical nature of the Miller scale may not fully capture the continuous relationship between lesion burden and outcome. Quantitative volumetric approaches and lesion-location mapping techniques have demonstrated stronger associations with developmental outcomes and may complement conventional grading in future clinical practice [39,41].

**Table 2:** MRI Scoring Systems for Preterm Infants.

Scoring System	Year	Original Cohort (n)	Summary of Scoring/Number of Items	Score Range	Imaging Timepoint	Estimated Time to Score/Ease of Use	Clinical vs. Research Tool	References
**Woodward**	2006	167	8 three-point scales: 5 WMI subscales (signal abnormality, PV WM volume loss, cystic abnormalities, ventricular dilatation, CC thinning) + 3 GMI subscales (signal abnormality, gyral maturation, subarachnoid space)	WMI 5–15; GMI 3–9	TEA	~10–15 min; moderate complexity	Clinical and research	[34]
**Kidokoro (GBAS)**	2013	97 preterm + 22 term controls	4 domains: WM, cortical GM, deep GM, cerebellum; combines injury + growth (biometric measurements including BPW, IHD, CC thickness, TCD, ventricular diameter, DGM area)	GBAS = sum of domain scores	TEA (validated also at 32 weeks PMA)	~15–20 min; moderate-high complexity	Clinical and research (most widely used)	[33,34,35,36]
**Miller WMI Classification**	2005	86	3 grades based on T1 punctate lesion number, size, and hemispheric extent: Grade 1 (≤3 foci, unilateral), Grade 2 (>3 foci or bilateral), Grade 3 (>3 foci extending beyond PV WM)	Grades 1–3	Early-life MRI (30–34 weeks PMA optimal)	~5 min; simple, quick categorical grading	Clinical and research	[38,39,40,41]
**Martinez-Biarge WMI**	2016/2019	82	4 grades of non-hemorrhagic WMI severity based on sequential imaging; emphasizes cyst evolution, PLIC myelination, and signal changes over time	Grades I–III	Sequential (early + TEA)	~10–15 min; requires sequential imaging comparison	Primarily research (emerging clinical)	[11,12]

### 10.7. The Martinez-Biarge WMI Classification

A four-grade system specifically for preterm non-hemorrhagic white matter injury, the Martinez-Biarge WMI classification provides significant information from sequential MRI analysis. In the original validation cohort of 82 preterm infants, WMI severity was correlated with the presence and severity of cerebral palsy [11]. For infants with grade I WMI, 9% developed a milder form cerebral palsy with achievement of walking at a later age. For grades II and III WMI, most children (grade II 83%; grade III 91%) developed cerebral palsy, with all having additional neurodevelopmental impairments [11]. For grade III WMI, children who did not develop cerebral palsy had white matter cysts that did not impact on the corticospinal tracts with normal myelination in the PLIC. This highlights the prognostic value of individualized assessment of lesion location and myelination progression [11]. This system emphasizes the importance of sequential imaging, as cysts may reduce in size or disappear by TEA, potentially underestimating injury severity on a single TEA scan [12].

### 10.8. IVH-Specific Scoring

The Papile classification, originally developed for CT interpretation and subsequently adapted for cranial ultrasonography, remains the most widely used grading system for germinal matrix–intraventricular hemorrhage (GM-IVH): grade I (subependymal hemorrhage), grade II (IVH occupying 10–50% of the ventricular area), grade III (IVH with >50% of ventricular area), and grade IV (parenchymal hemorrhagic venous infarction) [2,20]. Low-grade IVH (grades I–II) has historically been considered to have minimal long-term consequences; however, large geographic cohort studies have shown that even low-grade IVH is associated with a small increase in the risk of cerebral palsy and a marked increase in early cognitive delay and visual impairment [20]. High-grade IVH is associated with a substantially higher risk: children are six times as likely to have cerebral palsy, 11 times as likely to have visual impairment, and four times as likely to have bilateral hearing loss compared with those without IVH [20].

The conventional Papile grading system, however, does not capture the full complexity of brain injury associated with IVH. The Goeral IVH-specific MRI scoring system was developed specifically for preterm infants with IVH, consisting of 11 items assessed at TEA MRI that provide a comprehensive evaluation of important brain areas and potential additional abnormalities commonly associated with IVH [42]. This scoring system showed strong predictive capacity for outcomes at 2–3 years of age, with a translatable table for clinical use [42]. The Goeral system better represents the severity of brain damage compared with the conventional IVH classification, as it accounts for co-occurring injuries, such as white matter injury, cerebellar hemorrhage, and post-hemorrhagic ventricular dilatation, which significantly influence prognosis [42,43].

Brain MRI has additional value beyond cranial ultrasonography for detecting co-occurring subtle brain lesions in infants with IVH, including temporal and occipital hemorrhages, concurrent white matter injury, and cerebellar hemorrhage, all of which improve neuroprognostication [44]. Prognosis depends not only on the grade of GM-IVH but also on co-occurring preterm brain injuries and their downstream effects on brain maturation [43].

### 10.9. Scoring for Infants with Congenital Heart Disease

Infants with critical congenital heart disease (CHD) represent a distinct population within the neonatal population that can demonstrate white matter abnormalities similar to those of premature infants [45]. WMI occurs pre-operatively in approximately 20% of infants with complex CHD and is associated with brain immaturity, while new WMI after cardiac surgery has been found in >40% of infants, although recent data suggest this rate is declining with improvements in post-operative management [46]. The greatest risk for WMI occurs among those with single-ventricle heart disease with arch obstruction [46].

The brain injury severity (BIS) score is one proposed metric to understand and objectively assess injury in these infants. The BIS score categorizes injury on an ordinal scale: 0 = none or minimal injury (mild WMI defined as 1–3 foci each 2 mm, and IVH grade I–II; no stroke); 1 = stroke (any size stroke without moderate-to-severe WMI); and 2 = moderate-to-severe injury (moderate WMI defined as >3 foci or any foci > 2 mm, and severe WMI defined as >5% of white matter volume) [47]. The BIS score is assigned to both pre-operative and post-operative MRI for newly acquired lesions, with a maximal BIS score determined as the highest score between the two timepoints [47]. Moderate-to-severe degrees of WMI are independently associated with cognitive and motor outcomes in childhood [46].

A European multicenter collaboration proposed a standardized MRI protocol for CHD neonates that includes T1, T2, DWI, SWI, and MR venography to capture the full spectrum of ischemic, hemorrhagic, and thrombotic lesions [15]. Additional MRI scoring approaches for CHD neonates have been proposed that categorize findings into cognitive/gray matter, motor/white matter, and visual functional areas, with the MRI cognitive score showing direct correlation with respiratory index prior to surgery and cross-clamping time [48]. The 2024 American Heart Association scientific statement emphasizes that all patients with critical CHD lesions are at risk for impaired outcomes, and that developmental and genetic screening is indicated [46].

However, the indications for brain MRI in an asymptomatic child with CHD remain poorly defined given the unclear prognostic value of abnormal findings and the lack of consensus on the need for treatment of asymptomatic periventricular leukomalacia [45]. Unlike the white matter lesions found in premature infants, the white matter lesions of infants after cardiac surgery may no longer be detectable by routine MRI within months of the original findings, suggesting that more sensitive imaging techniques may be required to visualize white matter injury after the resolution of acute injury [45].

## 11. Applications in the Preterm and Term Infant: Outcomes and Clinical Impact

### 11.1. Prognostic Value of MRI in Term Neonates with Encephalopathy

MRI is the gold standard for characterizing brain injury related to neonatal encephalopathy and provides the most robust prognostic information when combined with advanced modalities [9]. In term neonates treated with therapeutic hypothermia, the pattern and severity of injury from MRI strongly predict neurodevelopmental outcomes. The basal ganglia–thalamus pattern of injury is associated with the most severe motor outcomes, including spastic quadriplegic cerebral palsy, while the watershed pattern is more commonly associated with cognitive deficits [5]. Brain MRI, performed as soon as possible after rewarming (days 4–5) or in the first week after birth, allows for optimal classification of injury based on DWI, while MRI at 10 days of life or later best delineates the full extent of cerebral injury on conventional sequences [6].

The combination of MRI scoring with quantitative biomarkers, ADC values, or lactate/NAA ratios in the basal ganglia has a better association with outcome than MRI scoring alone [9]. By standardizing acquisition protocols and post-processing, MRI biomarkers can serve as reliable, early surrogate endpoints in neuroprotection trials, allowing smaller sample sizes and accelerating clinical translation [9].

### 11.2. Prognostic Value of MRI in Preterm Neonates

In preterm infants, the prognostic value of TEA MRI is well-established but nuanced. Abnormal findings on MRI at TEA in infants born at 30 weeks’ gestation have been shown to be predictive of psychomotor delay and cerebral palsy at 2 years of age [2]. The association with adverse neurodevelopmental outcome at 7 years of age was particularly striking for abnormalities in the white matter, deep gray matter, and cerebellum [2,37]. In a large, unselected cohort of 504 preterm infants imaged at TEA, 76% had acquired lesions. Abnormal motor outcomes were found in all infants with periventricular leukomalacia and 60% of those with hemorrhagic parenchymal infarction [49]. However, limited sensitivity and specificity were documented for normal outcomes [49]. The relationship and reliability of prediction of neurodevelopmental outcomes may in part relate to the nature of the scoring system and evaluative measures of the MRI scan.

Emerging evidence suggests that early-life MRI (acquired at 29–35 weeks postmenstrual age) may provide complementary or even superior prognostic information compared with TEA MRI for certain injury types. Moderate–severe and anterior white matter injury on early-life MRI (but not TEA MRI) was associated with cognitive delay (OR 3.28) and motor delay (OR 3.02) at 36 months in very preterm infants [12]. Early brain morphometrics from neonatal MRI predicted motor and cognitive outcomes at 2 years corrected age, with an AUC of 0.86 for both scores [16]. These findings support the utility of imaging prior to TEA for earlier commencement of targeted interventions.

Combining imaging and clinical risk factors is important for risk stratification of preterm infants. Even mild brain injuries, such as IVH grade II, contribute to the risk of adverse outcomes, underscoring the importance of comprehensive MRI assessments [1]. Independent key risk factors for adverse outcomes include the presence of more than one severe MRI-detected brain injury, a low 10 min Apgar score, and the number of red blood cell transfusions received [1].

### 11.3. Timing of MRI and Clinical Decision-Making

The optimal timing for neonatal brain MRI remains an area of active investigation. For term neonates with encephalopathy, brain MRI can be performed with complexity during therapeutic hypothermia (beyond the first 24 h) if information is needed to inform goals-of-care decisions. To undertake imaging during therapeutic hypothermia requires either a compatible in-NICU MR system or the temporary removal of the hypothermia device with monitoring of the infant’s temperature only to prevent warming. These are both challenging. Thus, in clinical practice, optimal injury detection usually occurs with DWI at days 3–5, and the full extent of injury is best delineated at approximately 10 days [6]. For preterm infants, the AAP recommends that MRI is not indicated as a routine procedure but may be offered at TEA to high-risk infants after discussion with families regarding the limitations of this test for estimation of long-term prognosis [2]. When possible, brain MRI should always be performed without contrast in a non-sedated state using a “feed-and-wrap” technique [2].

Controversy persists regarding the clinical utility of routine TEA MRI in preterm infants. Some studies have reported that adding MRI to early and late cranial ultrasonography does not improve the prediction of severe intellectual disability or neurodevelopmental impairment at 6–7 years of age [2]. Obtaining routine MRI appeared to slightly reduce maternal anxiety, even though it may increase the cost of care [2]. Regardless, MRI remains superior to cranial ultrasonography for detecting WMI, cerebellar hemorrhage, and other abnormalities that may inform parental counseling and the allocation of early intervention services [50]. There is also information to suggest that the specificity of MRI findings can also direct the quality and focus of in-patient and out-patient therapies, which may substantially change the trajectory of recovery in preterm infants.

### 11.4. Early Intervention and Family Counseling

The ultimate clinical value of neonatal brain MRI lies in its ability to guide early intervention and inform family counseling. Early accurate identification of infants at risk of adverse neurodevelopmental outcomes enables prognostication and initiation of early targeted interventions during the period of highest brain plasticity [16,51]. The development of sophisticated MRI strategies, including DTI, resting-state functional connectivity, and MRS, may increase the prognostic value of neonatal neuroimaging, helping to guide parental counseling and allocate early intervention services [52].

However, MRI results’ relationship to outcomes may not be predictable for an individual family, and cautious counseling is essential, particularly for mild and moderate injury where outcomes are highly variable [2]. For children with milder forms of brain injury, environmental factors such as the level of maternal education become increasingly important for cognitive development as children grow older, suggesting that the social environment may modulate the impact of early brain injury on long-term outcomes [33].

## 12. Limitations and Future Directions

### 12.1. Current Limitations

Despite the remarkable advances in neonatal brain MRI, several important limitations persist. The predictive accuracy of MRI for individual patients remains imperfect, particularly for mild and moderate injury where outcomes are highly variable [7,49]. The explained variance of TEA MRI scores for neurodevelopmental outcomes is often low (R^2^ ≤ 0.219 in some cohorts), reflecting the multifactorial nature of neurodevelopmental trajectories [35]. The patterns of brain injury may differ between cohorts and centers, limiting the generalizability of scoring systems [35]. Additionally, the neonatal brain’s rapid developmental changes create a moving target for interpretation, and normative reference data remain limited for many advanced MRI metrics.

Practical challenges include the need for specialized equipment, trained personnel, and age-specific acquisition protocols. Motion artifacts remain a significant concern, and while feed-and-wrap techniques are generally successful, some infants require sedation, which carries its own risks [2]. Access to MRI varies considerably across institutions and geographic regions, creating disparities in the availability of advanced neuroimaging for high-risk neonates [44].

### 12.2. Emerging Technologies

Low-field and portable MRI systems represent a major advancement in neonatal neuroimaging. Dedicated low-field MRI systems within the NICU and trials of ultra-low-field portable MRI systems at the bedside are being developed, potentially eliminating the need to transport critically ill neonates to the radiology suite [44]. These systems may democratize access to brain MRI for neonates in resource-limited settings.

Ultrafast brain MRI techniques are being employed to decrease image acquisition time, reducing the window for motion artifact and improving feasibility in unsedated neonates [44]. Higher-field magnets (3T and beyond) are being utilized to enhance image quality and signal-to-noise ratio, enabling more detailed assessment of subtle structural abnormalities [44].

### 12.3. Artificial Intelligence and Machine Learning

The application of artificial intelligence (AI) and machine learning (ML) to neonatal brain MRI represents a promising future direction. Deep-learning-based frameworks have demonstrated the ability to distinguish normal from abnormal neonatal brain scans with up to 83% accuracy, and critically, have identified new brain anomalies originally missed during radiological reading [53]. Automated neuroprognostication using ML models incorporating MRI-based measures has shown promise in one study, where it was shown to predict 18-month Bayley scores in neonates with HIE, with correlations between predicted and observed outcomes reaching 0.94 and predictive R^2^ of 0.87 across cognitive, language, and motor domains [54]. These models may help predict outcomes across the full spectrum of injury severity, not just in severe cases, but require more extensive investigation and validation in many research and clinical settings [55]. 

Deep-learning-based pipelines for automated neonatal brain segmentation have helped to enable rapid and reproducible volumetric analysis. Three-dimensional neural networks have been applied to detect subcortical brain dysmaturation in CHD [56]. Deep neural networks trained on structural T2-weighted images may help predict postmenstrual age at scan, with a mean absolute error of only 0.46 weeks, providing an objective measure of brain maturation [57].

Emerging AI/ML tools may create opportunities for enhanced prognostication through multimodal analyses that integrate neuroimaging with clinical, electrophysiological, and biomarker data [58]. Neuro-multimodal monitoring strategies combining MRI with EEG, near-infrared spectroscopy, and clinical variables demonstrate superior accuracy compared with single-modality approaches for both injury identification and prediction of neurodevelopmental outcomes [59].

However, several challenges remain, including insufficient standardization of monitoring parameters, limited multicenter data sharing, and the “black-box” nature and poor clinical interpretability of AI algorithms, which hinder clinical translation [59]. The lack of transparency and regulatory neonatal frameworks in AI algorithms can be problematic when applying conclusions and interpretations to clinical care [60]. Furthermore, these algorithms are particularly constrained by adequate sample size and data; given neonatal datasets are particularly limited, this can further impact accuracy and generalizability of output [61]. It is unclear what role algorithmic bias may also have, given that many datasets involve primarily White, North American, and European populations, which can exacerbate existing health inequities [61]. Implementation costs can also offer further challenges in widespread use [62].

## 13. Conclusions

MRI has transformed the understanding of neonatal brain injury and development, providing clinicians with an unprecedented array of tools for diagnosis, prognostication, and therapeutic decision-making. Conventional T1 and T2 imaging remains the foundation for structural assessment, while DWI with ADC mapping enables early detection of acute injury. DTI and advanced diffusion techniques provide detailed characterization of white matter microstructures and developmental trajectories. MRS offers unique metabolic information with robust prognostic value, particularly the lactate/NAA ratio and thalamic NAA concentration. SWI enhances hemorrhage detection, volumetric analysis quantifies brain growth and dysmaturation, ASL assesses cerebral perfusion, and functional connectivity MRI reveals the emerging architecture of brain networks.

Validated scoring systems, including the NICHD, Weeke, and Trivedi systems for term encephalopathy and the Kidokoro, Woodward, and Martinez-Biarge systems for preterm injury, provide standardized frameworks for injury quantification and outcome prediction. The deep gray matter subscore consistently emerges as the strongest predictor of adverse outcomes across scoring systems in term infants, while white matter and cerebellar abnormalities carry particular prognostic significance in preterm populations.

The fundamental differences between preterm and term neonatal brain injury, in terms of etiology, pattern, imaging characteristics, and developmental contexts, necessitate population-specific approaches to MRI interpretation and scoring. The concept of brain dysmaturation, distinct from but still overlapping with brain injury, has emerged as a critical framework for understanding the neurologic consequences of preterm birth. Future advances in portable MRI technology, artificial intelligence, and multimodal integration hold promise for improving access, accuracy, and clinical utility of neonatal brain imaging, ultimately enabling earlier and more targeted interventions to optimize neurodevelopmental outcomes in this vulnerable population.

## Figures and Tables

**Table 1:** MRI Scoring Systems for Term Infants.

Scoring System	Year	Original Cohort (n)	Summary of Scoring/Number of Items	Score Range	Estimated Time to Score/Ease of Use	Clinical vs. Research Tool	References
**Barkovich**	1998	30	4 scoring categories: BG score, watershed (W) score, combined BG/W score, summation (S) score; assessed on T1, T2 sequences	0–11 (modified)	~5 min; straightforward, pattern-based	Clinical and research	[28]
**Rutherford**	2010	56	4 items: deep gray matter (0–3), white matter (0–3), cortex (0–3), PLIC signal (0–2); total injury score	0–11	~5–10 min; moderate complexity	Clinical and research	[28]
**NICHD NRN**	2005 (expanded 2025)	208 (original); 382 (expanded)	Ordinal scale: 0 (normal) through 3 (hemispheric devastation); expanded version adds laterality and separates WS from BGT/PLIC injury	0–3 (original); 0–2B3/3 (expanded)	~5 min; simple categorical assignment	Clinical and research	[28]
**Weeke**	2018	97 (cohort 1); 76 (cohort 2)	3 subscores: deep gray matter, white matter/cortex, cerebellum; includes DWI assessment; most comprehensive	Continuous (sum of subscores)	~10–15 min; moderate-high complexity	Primarily research (emerging clinical)	[28]
**Trivedi**	2017	81	Categorical system assessing BGT, WM, cortex; simplified grading	Categorical (normal to severe)	~5 min; simple categorical	Clinical and research	[28]

## Data Availability

No new data were created or analyzed in this study. Data sharing is not applicable to this article.

## References

[1] Drommelschmidt K., Mayrhofer T., Müller H., Foldyna B., Raudzus J., Göricke S., Schweier B., Sirin S. (2025). CMRI-detected Brain Injuries and Clinical Key Risk Factors Associated with Adverse Neurodevelopmental Outcomes in Very Preterm Infants. Sci. Rep..

[2] Hand I.L., Shellhaas R.A., Milla S.S. (2020). Routine Neuroimaging of the Preterm Brain. Pediatrics.

[3] Dubois J., Alison M., Counsell S.J., Hertz-Pannier L., Hüppi P., Benders M. (2021). MRI of the Neonatal Brain: A Review of Methodological Challenges and Neuroscientific Advances. J. Magn. Reson. Imaging.

[4] Handa A., Xu L., Machado-Rivas F., Cortex-Albornoz M., Ruggiero M., Bendoya M., Yang E., Gee M., Jaimes C. (2023). Magnetic Resonance Imaging in Neonates: A Practical Approach to Optimize Image Quality and Increase Diagnostic Yield. Pediatr. Radiol..

[5] Douglas-Escobar M., Weiss M.D. (2015). Hypoxic-Ischemic Encephalopathy: A Review for the Clinician. JAMA Pediatr..

[6] Zanelli S.A., Wusthoff C.J., Lucke A.M., Kaufman D. (2026). Therapeutic Hypothermia for Neonatal Hypoxic-Ischemic Encephalopathy: Clinical Report. Pediatrics.

[7] Shankaran S., Laptook A.R., Guimaraes C., Murnick J., McDonald S., Das A., Huitema C., Pappas A., Higgins R., Hintz S. (2025). NICHD Magnetic Resonance Brain Imaging Score in Term Infants with Hypoxic-Ischemic Encephalopathy: A Secondary Analysis of a Randomized Clinical Trial. JAMA Pediatr..

[8] Groenendaal F., de Vries L.S. (2017). Fifty Years of Brain Imaging in Neonatal Encephalopathy Following Perinatal Asphyxia. Pediatr. Res..

[9] Laptook A., Garvey A.A., Adams C., Grant P.E., Molloy E.J., Groenendaal F., Weeke L.C., Benders M., Hwang M., El-Dib M. (2025). Magnetic Resonance Imaging and Spectroscopy in Neonatal Encephalopathy: Current Consensus Position and Future Opportunities. Pediatr. Res..

[10] American College of Obstetricians and Gynecologists (2014). Executive Summary: Neonatal Encephalopathy and Neurologic Outcome, Second Edition. Report of the American College of Obstetricians and Gynecologists’ Task Force on Neonatal Encephalopathy. Obstet. Gynecol..

[11] Martinez-Biarge M., Groenendaal F., Kersbergen K.J., Bender M., Foti F., Haastert I., Cowan F., de Vries L.S. (2019). Neurodevelopmental Outcomes in Preterm Infants with White Matter Injury Using a New MRI Classification. Neonatology.

[12] Selvanathan T., Oloomi S., Guo T., Ufkes S., Chau V., Branson H., Synnes A., Ly L., Kelly E., de Vries L.S. (2025). Association of Early-Life or Term-Equivalent White Matter Injury with Neurodevelopmental Outcomes in Very Preterm Infants. Neurology.

[13] Dibble M., Ang J.Z., Mariga L., Molloy E.J., Bokde A.L.W. (2021). Diffusion Tensor Imaging in Very Preterm, Moderate-Late Preterm and Term-Born Neonates: A Systematic Review. J. Pediatr..

[14] Onda K., Chavez-Valdez R., Graham E.M., Everett A., Northington F., Oishi K. (2024). Quantification of Diffusion Magnetic Resonance Imaging for Prognostic Prediction of Neonatal Hypoxic-Ischemic Encephalopathy. Dev. Neurosci..

[15] Stegeman R., Feldmann M., Claessens N.H.P., Jansen N.J.G., Breur J.M.P.J., de Vries L.S., Logeswaran T., Reich B., Knirsch W., Kottke R. (2021). A Uniform Description of Perioperative Brain MRI Findings in Infants with Severe Congenital Heart Disease: Results of a European Collaboration. AJNR Am. J. Neuroradiol..

[16] Pagnozzi A.M., van Eijk L., Pannek K., Boyd R.N., Saha S., George J., Bora S., Bradford D., Fahey M., Ditchfield M. (2023). Early Brain Morphometrics From Neonatal MRI Predict Motor and Cognitive Outcomes at 2-Years Corrected Age in Very Preterm Infants. NeuroImage.

[17] Beare R.J., Chen J., Kelly C.E., Alexopoulos D., Smyser C.D., Rogers C.E., Loh W.Y., Matthews L.G., Cheong J.L., Spittle A.J. (2016). Neonatal Brain Tissue Classification with Morphological Adaptation and Unified Segmentation. Front. Neuroinform..

[18] Romberg J., Wilke M., Allgaier C., Nägele T., Engel C., Poets C.F., Franz A. (2022). MRI-based Brain Volumes of Preterm Infants at Term: A Systematic Review and Meta-Analysis. Arch. Dis. Child.-Fetal Neonatal Ed..

[19] Hüppi P.S., Warfield S., Kikinis R., Barnes P.D., Zientara G.P., Jolesz F.A., Tsuji M.K., Volpe J.J. (1998). Quantitative Magnetic Resonance Imaging of Brain Development in Premature and Mature Newborns. Ann. Neurol..

[20] Inder T.E., Volpe J.J., Anderson P.J. (2023). Defining the Neurologic Consequences of Preterm Birth. N. Engl. J. Med..

[21] Shen D.D., Bao S.L., Wang Y., Chen Y.C., Zhang Y.C., Li X.C., Ding Y.C., Jia Z.Z. (2023). An Automatic and Accurate Deep Learning-Based Neuroimaging Pipeline for the Neonatal Brain. Pediatr. Radiol..

[22] Iutaka T., de Freitas M.B., Omar S.S., Scortegagna F.A., Nael K., Nunes R.H., Pacheco F.T., Júnior A.C.M.M., do Amaral L.L.F., Rocha A.J. (2023). Arterial Spin Labeling: Techniques, Clinical Applications, and Interpretation. Radiographics.

[23] Delmas J., Toupin S., Pfeuffer J., Chateil J.F. (2022). A Practical Guide to Optimize Arterial Spin Labeling in Neonates at 1.5 Tesla: What the Radiologist Needs to Know. Pediatr. Radiol..

[24] Tortora D., Severino M., Rossi A. (2020). Arterial Spin Labeling Perfusion in Neonates. Semin. Fetal Neonatal Med..

[25] Narayanan S., Schmithorst V., Panigrahy A. (2020). Arterial Spin Labeling in Pediatric Neuroimaging. Semin. Pediatr. Neurol..

[26] Prysiazhniuk Y., Alexander S., Armindo R.D., Tong E., Yeom K.W., Otáhal J., Kynčl M., Moseley M., Petr J., Zhao M. (2025). Comparative Evaluation of Single- and Multi-Delay Arterial Spin Labeling MRI in Preterm Neonates. NeuroImage.

[27] Hu Z., Jiang D., Shepard J., Uchida Y., Oishi K., Shi W., Liu P., Lin D., Yedavalli V., Tekes A. (2025). High-Fidelity MRI Assessment of Cerebral Perfusion in Healthy Neonates Less Than 1 Week of Age. J. Magn. Reson. Imaging.

[28] Finnegan E., Assi A., Carroll E., Jennings A.H., Roe D., Olaseine O., McCarthy A., Wong Y.J., O’Connor N., Dalton C. (2025). MRI Scoring Systems in Neonatal Encephalopathy and Neurodevelopmental Outcomes: A Systematic Review. J. Perinatol..

[29] Andorka C., Barta H., Sesztak T., Nyilas N., Kovacs K., Dunai L., Rudas G., Jermendy A., Szabo M., Szakmar E. (2025). The Predictive Value of MRI Scores for Neurodevelopmental Outcome in Infants with Neonatal Encephalopathy. Pediatr. Res..

[30] Wu Y.W., Monsell S.E., Glass H.C., Wisnowski J., Mathur A., McKinstry R., Bluml S., Gonzalez F.F., Comstock B., Heagerty P. (2023). How Well Does Neonatal Neuroimaging Correlate with Neurodevelopmental Outcomes in Infants with Hypoxic-Ischemic Encephalopathy?. Pediatr. Res..

[31] Spencer A.P.C., Lequin M.H., de Vries L.S., Brooks J.C.W., Jary S., Tonk J., Cowan F.M., Thorensen M., Chakkarapani E. (2023). Mammillary Body Abnormalities and Cognitive Outcomes in Children Cooled for Neonatal Encephalopathy. Dev. Med. Child Neurol..

[32] Annink K.V., de Vries L.S., Groenendaal F., Eijsermans R.M., Mocking M., van Schooneveld M.M., Dudink J., van Straaten H.L., Benders M.J., Lequin M. (2021). Mammillary Body Atrophy and Other MRI Correlates of School-Age Outcome Following Neonatal Hypoxic-Ischemic Encephalopathy. Sci. Rep..

[33] Jansen L., van Steenis A., van den Berg-Huysmans A.A., Wiggers-de Bruine S.T., Rijken M., de Vries L.S., Vermeiren R.R.J.M., Peeters-Scholte C.M.P.C.D., Steggerda S.J. (2021). Associations Between Neonatal Magnetic Resonance Imaging and Short- and Long-Term Neurodevelopmental Outcomes in a Longitudinal Cohort of Very Preterm Children. J. Pediatr..

[34] Woodward L.J., Anderson P.J., Austin N.C., Howard K., Inder T.E. (2006). Neonatal MRI to Predict Neurodevelopmental Outcomes in Preterm Infants. N. Engl. J. Med..

[35] Brouwer M.J., Kersbergen K.J., van Kooij B.J.M., Benders M.J.N.L., van Haastert I.C., Koopman-Esseboom C., Neil J.J., de Vries L.S., Kidokoro H., Inder T.E. (2016). Preterm Brain Injury on Term-Equivalent Age MRI in Relation to Perinatal Factors and Neurodevelopmental Outcome at Two Years. PLoS ONE.

[36] Bonezzi L., Biagioni T., Fiori S., Luke C., George J.M., Colditz P.B., Fripp J., Ware R.S., Pannek K., Boyd R.N. (2026). Concordance and Association of Kidokoro MRI Scores at 32 and 40 Weeks Post-Menstrual Age with Neurodevelopmental Outcomes in Very Preterm Infants. AJNR Am. J. Neuroradiol..

[37] Anderson P.J., Treyvaud K., Neil J.J., Cheong J.L.Y., Hunt R.W., Thompson D.K., Lee K.J., Doyle L.W., Inder T.E. (2017). Associations of Newborn Brain Magnetic Resonance Imaging with Long-Term Neurodevelopmental Impairments in Very Preterm Children. J. Pediatr..

[38] Cayam-Rand D., Guo T., Grunau R.E., Benavente-Fernández I., Synnes A., Chau V., Branson H., Latal B., McQuillen P., Miller S.P. (2019). Predicting Developmental Outcomes in Preterm Infants: A Simple White Matter Injury Imaging Rule. Neurology.

[39] Guo T., Duerden E.G., Adams E., Chau V., Branson H.M., Chakravarty N.M., Poskitt K.J., Synnes A., Grunau R.E., Miller S.P. (2017). Quantitative Assessment of White Matter Injury in Preterm Neonates: Association with Outcomes. Neurology.

[40] Tusor N., Benders M.J., Counsell S.J., Nongena P., Ederies M.A., Falconer S., Chew A., Gonzalez-Cinca N., Hajinal J.V., Gangadharan S. (2017). Punctate White Matter Lesions Associated with Altered Brain Development and Adverse Motor Outcome in Preterm Infants. Sci. Rep..

[41] Selvanathan T., Guo T., Ufkes S., Chau V., Branson H., Synnes A., Ly L.G., Kelly E.N., Grunau R.E., Miller S.P. (2024). Size and Location of Preterm Brain Injury and Associations with Neurodevelopmental Outcomes. Neurology.

[42] Goeral K., Kasprian G., Hüning B.M., Waldhoer T., Fuiko R., Schmidbauer V., Prayer D., Felderhoff-Müser U., Berger A., Olischar M. (2022). A Novel Magnetic Resonance Imaging-Based Scoring System to Predict Outcome in Neonates Born Preterm with Intraventricular Haemorrhage. Dev. Med. Child Neurol..

[43] Leijser L.M., de Vries L.S., Cizmeci M.N. (2026). Long-Term Neurodevelopmental Outcomes and Follow-Up of Germinal Matrix-Intraventricular Hemorrhage in Preterm Infants. Semin. Fetal Neonatal Med..

[44] Cizmeci M.N., Leijser L.M., de Vries L.S. (2026). Diagnostic Neuroimaging and Early Detection of Germinal Matrix Hemorrhage-Intraventricular Hemorrhage. Semin. Fetal Neonatal Med..

[45] Marino B.S., Lipkin P.H., Newburger J.W., Peacock G., Gerdes M., Gayor J.W., Mussatto K.A., Uzark K., Goldberg C.S., Johnson W.H. (2012). Neurodevelopmental Outcomes in Children with Congenital Heart Disease: Evaluation and Management: A Scientific Statement From the American Heart Association. Circulation.

[46] Sood E., Newburger J.W., Anixt J.S., Cassidy A.R., Jackson J.L., Jonas R.A., Lisanti A.J., Keila N.L., Peyvandi S., Marino B.S. (2024). Neurodevelopmental Outcomes for Individuals with Congenital Heart Disease: Updates in Neuroprotection, Risk-Stratification, Evaluation, and Management: A Scientific Statement From the American Heart Association. Circulation.

[47] Peyvandi S., Chau V., Guo T., Xu D., Glass H., Synnes A., Poskitt K., Barkovich A.J., Miller S.P., McQuillen P.S. (2018). Neonatal Brain Injury and Timing of Neurodevelopmental Assessment in Patients with Congenital Heart Disease. J. Am. Coll. Cardiol..

[48] Bhattacharjee I., Mohamed M.A., Nandakumar V., Friedman N.R., Ruggieri P., Aly H. (2022). Scoring of Brain Magnetic Resonance Imaging and Neurodevelopmental Outcomes in Infants with Congenital Heart Disease. Early Hum. Dev..

[49] Arulkumaran S., Tusor N., Chew A., Falconer S., Kennea N., Nongena P., Hajnal J.V., Counsell S.J., Rutherford M.A., Edwards A.D. (2020). MRI Findings at Term-Corrected Age and Neurodevelopmental Outcomes in a Large Cohort of Very Preterm Infants. AJNR Am. J. Neuroradiol..

[50] Ibrahim J., Mir I., Chalak L. (2018). Brain Imaging in Preterm Infants 32 Weeks Gestation: A Clinical Review and Algorithm for the Use of Cranial Ultrasound and Qualitative Brain MRI. Pediatr. Res..

[51] Harmony T. (2021). Early Diagnosis and Treatment of Infants with Prenatal and Perinatal Risk Factors for Brain Damage at the Neurodevelopmental Research Unit in Mexico. NeuroImage.

[52] Kwon S.H., Vasung L., Ment L.R., Huppi P.S. (2014). The Role of Neuroimaging in Predicting Neurodevelopmental Outcomes of Preterm Neonates. Clin. Perinatol..

[53] Raad J.D., Chinnam R.B., Arslanturk S., Tan S., Jeong J.W., Mody S. (2023). Unsupervised Abnormality Detection in Neonatal MRI Brain Scans Using Deep Learning. Sci. Rep..

[54] Lewis J.D., Miran A.A., Stoopler M., Branson H.M., Danguecan A., Raghu K., Ly L.G., Cizmeci M.N., Kalish B.T. (2026). Neuroprognostication via Radiomics and Machine Learning Following Neonatal Hypoxic-Ischemic Insult. J. Pediatr..

[55] Lewis J.D., Miran A.A., Stoopler M., Branson H.M., Danguecan A., Raghu K., Ly L.G., Cizmeci M.N., Kalish B.T. (2025). Automated Neuroprognostication via Machine Learning in Neonates with Hypoxic-Ischemic Encephalopathy. Ann. Neurol..

[56] Ceschin R., Zahner A., Reynolds W., Gaesser J., Zuccoli G., Lo C.W., Gopalakrishnan V., Panigrahy A. (2018). A Computational Framework for the Detection of Subcortical Brain Dysmaturation in Neonatal MRI Using 3D Convolutional Neural Networks. NeuroImage.

[57] Beizaee F., Bona M., Desrosiers C., Dolz J., Lodygensky G. (2023). Determining Regional Brain Growth in Premature and Mature Infants in Relation to Age at MRI Using Deep Neural Networks. Sci. Rep..

[58] Chawla V., Cizmeci M.N., Sullivan K.M., Gritz E., Cardona V.Q., Menkiti O., Natarajan G., Rao R., McAdams R.M., Dizon M.L. (2025). Emerging Modalities for Neuroprognostication in Neonatal Encephalopathy: Harnessing the Potential of Artificial Intelligence. Pediatr. Res..

[59] Wang J., Yan Y., Shen Y., Zhang C., Du F., Luo M., Zhao S., Zhang R., Dong W. (2025). Neuro-Multimodal Monitoring in Neonatal Brain Injury: Current Applications, Challenges, and Future Directions. Brain Res. Bull..

[60] Kwok T.C., Henry C., Saffaran S., Meeus M., Bates D., Van Laere D., Boylan G., Boardman J.P., Sharkey D. (2022). Application and Potential of Artificial Intelligence in Neonatal Medicine. Semin. Fetal Neonatal Med..

[61] Speiser J.L., Kerr W.T., Ziegler A. (2024). Common Critiques and Recommendations for Studies in Neurology Using ML Methods. Neurology.

[62] Arora T., Muhammad-Kamal H., Beam K. (2025). Preserving Medical Ethics in the Era of Artificial Intelligence: Challenges and Opportunities in Neonatology. Semin. Perinatol..

